# Deep Phenotyping of Coarse Root Architecture in *R.* *pseudoacacia* Reveals That Tree Root System Plasticity Is Confined within Its Architectural Model

**DOI:** 10.1371/journal.pone.0083548

**Published:** 2013-12-27

**Authors:** Frédéric Danjon, Hayfa Khuder, Alexia Stokes

**Affiliations:** 1 INRA, UMR1202 BIOGECO, Cestas, France; 2 Université de Bordeaux, UMR1202 BIOGECO, Cestas, France; 3 Université de Bordeaux, UMR 5295, Institut de Mécanique et d'Ingénierie - Bordeaux (I2M), Département Génie Civil et Environnemental (GCE), Bordeaux, France; 4 INRA, UMR AMAP (Botanique et bioinformatique de l’architecture des plantes), Montpellier, France; University of Nottingham, United Kingdom

## Abstract

This study aims at assessing the influence of slope angle and multi-directional flexing and their interaction on the root architecture of *Robinia pseudoacacia* seedlings, with a particular focus on architectural model and trait plasticity. 36 trees were grown from seed in containers inclined at 0° (control) or 45° (slope) in a glasshouse. The shoots of half the plants were gently flexed for 5 minutes a day. After 6 months, root systems were excavated and digitized in 3D, and biomass measured. Over 100 root architectural traits were determined. Both slope and flexing increased significantly plant size. Non-flexed trees on 45° slopes developed shallow roots which were largely aligned perpendicular to the slope. Compared to the controls, flexed trees on 0° slopes possessed a shorter and thicker taproot held in place by regularly distributed long and thin lateral roots. Flexed trees on the 45° slope also developed a thick vertically aligned taproot, with more volume allocated to upslope surface lateral roots, due to the greater soil volume uphill. We show that there is an inherent root system architectural model, but that a certain number of traits are highly plastic. This plasticity will permit root architectural design to be modified depending on external mechanical signals perceived by young trees.

## Introduction

The way in which a coarse root system is established spatially in a tree determines both its anchorage and its capacity to absorb water and nutrients [1]. The framework of structural roots defines the position of absorbing roots and thus the conduction of water and nutrients, especially when soil resources are unevenly distributed [2], [3]. Understanding how plasticity in morphological and topological root traits is affected by environmental constraints is thus primordial if we are to better understand root system function and ecology. It is therefore necessary to improve analyses of root system architecture (geometry and topology) to better determine fundamental belowground processes.

Coarse root system architecture has often been assessed by measuring a limited number of characteristics using topological analysis [4], [5], fractal branching analysis [6], three-dimensional (3D) fractal dimension assessment [7] or root cross sectional area measurement at a fixed distance [8]. Recent methods for the coding, digitizing and analysis of root system topology and geometry provide a detailed and rapid assessment of differences in root architecture as a function of any given treatment [9]–[12]. Root architecture in larger potted plants [13] can now also be tracked efficiently in 3D using non-invasive techniques [14]. The large amount of data subsequently produced can be used to compute the 3D spatial distribution of root volume (e.g. [15]). However, root systems are largely structured by root type [16], therefore an analysis assessing characteristics and distribution by root type i.e. an “architectural analysis” [17]–[19] has proved to be more powerful. Nevertheless, even the most recent studies have not performed a deep phenotyping, i.e. a full analysis of all features of 3D architecture of individual root systems.

The structural root system of trees is laid down when trees are juvenile [20]. Aside from nutrient and water supply, directional primary growth and secondary thickening are growth processes influenced by external mechanical loading, e.g. dynamic wind loading [21] or substrate movement [22]. The way in which plants respond to dynamic mechanical perturbation (MP) on shoots, has been well-documented over several decades [23]–[25]. The term thigmomorphogenesis is widely used to describe such plant responses to MP [23], [24]. In woody plants, as a result of MP, stems are often shorter and thicker, but more flexible, allowing plants to bend without breaking. In trees subjected to unilateral wind loading, through either controlled experiments or when growing in a prevailing wind, significant modifications and asymmetry occur in root systems. Changes in taproot length [26], stump and lateral root radial growth [27] occur and lateral root topology is altered, resulting in a higher rate of branching per unit of soil on the windward sides of young trees [5]. This root system asymmetry is due to the selective reinforcement of those compartments most important for stability [17], [18]. However, in hilly regions, the crown and stems of trees may be mechanically loaded in several directions, due to e.g. simultaneous wind and snow loading, especially when growing on steep slopes [28]. Apart from biomass measurements [29], [30], the response of tree root systems to a multi-directional flexing of shoots has never been measured. In several experiments, plant material comprised nursery transplants where the root architecture at the end of the experiment was likely influenced by the initial architecture of the transplant and thus also affected by transplanting stress [31].

There is an increasing interest in understanding how tree root systems develop on hillslopes, largely because the diverse mechanical loads present can have consequences for the mechanical integrity of a tree, as well as the substrate in which it is anchored [1]. The influence of sloping terrain on root architecture is not well documented [32], although the architecture of coarse root systems can influence significantly slope stability [33]. Studying the increase in shear resistance of soil planted with different woody species, [34] showed that the combined effect of several root traits on shear resistance was greater than the simple sum of resistance conferred by individual traits. Therefore only a global analysis of root architecture can allow an understanding of how a tree responds to one or several processes occurring simultaneously.

Previous studies of tree root development on steep slopes give contrary results [15], due to differences in methodology and the fact that many different parameters, other than slope angle, influence root distribution [32]. Whereas the taproots of most tree species are positively gravitropic, surface lateral roots of certain conifers are diagravitropic, i.e. roots undergo initial upward growth, following the soil surface even when it is sloping [35]. However, when a plant has grown from seed on a slope, and no other environmental conditions e.g. light, water or nutrition are limiting, and when no other mechanical stress e.g. wind loading, exists, the only imposed stress on the root system will be the weight of the soil or the shoot and branches [36], and the geometry of the substrate. Nevertheless, such conditions rarely exist in a natural environment, especially on hillslopes where mechanical loading is frequent and superficial soil movement can occur [22]. Studying three tree species in an elfin forest growing on steep slopes (20 to 50°), and exposed to windspeeds of up to 24 m.s^−1^ parallel to the slope, [37] showed that 90% of the trees had a preferential upslope development of roots.

The disparity in results of different studies show that it is necessary to distinguish and separate the effect of substrate geometry, the effect of MP and their interaction, and determine which stress results in which plastic response. Such an experiment has not yet been performed.

We chose to study the interaction between dynamic mechanical loading (flexing) and soil geometry (slope) in seedlings of *Robinia pseudoacacia* L. It has been suggested that this species is useful for reinforcing soil on unstable or eroding slopes [38], [39]. An experiment was carried out in a greenhouse where seedlings of *R. pseudoacacia* were grown from seeds in containers at 0° and 45° and their shoots were subjected to multi-directional flexing. After 23 weeks, 3D root architecture was measured *in situ*. A deep phenotyping was performed by defining six root segment types, and also using topological and fractal branching analyses, which have not been previously used in combination.

We hypothesize that (1) in plants on a slope without flexing, changes only in the direction of root growth occur, with shallow roots growing parallel to the soil surface (2) flexing in plants growing on a 0° slope results in modifications in stump, taproot and lateral root size (3) flexing in plants growing on a 45° slope should induce additive effects, whereby upslope root volume, length and branching are increased. Results are discussed with regard to the use of deep phenotyping to unravel the differences in root trait plasticity and architectural model.

## Materials and Methods

Seeds of *R. pseudoacacia* (Vilmorin seed, Pusztavacs provenance, Hungary) were sown in a mix of local forest soil, composted bark and turf in 36 square pots (0.30 width×0.15 m depth) in a glasshouse. Two seeds were placed into the centre of each pot. Soil thickness in the pots averaged 0.11 m. Half the pots were tilted at 0° (controls), the other half at 45° (slope) in two randomised blocks. Air temperature was 22°C during the day and 12°C at night. Plants were directly illuminated from above with halogen lamps (314–494 µmol.m^−2^.s^−1^) from 06∶00 to 24∶00. Relative humidity was constant at 80%. Plants were watered daily using a fine spray to avoid damaging the soil surface. Three weeks after seedling germination, one plant from each container was removed through cutting the stem at the soil surface using scissors, seedlings were 15 cm height. The flexing treatment then began. Half the plants were randomly assigned to the flexing treatment and the remainder were used as controls. Using a bamboo rod, the top of seedlings were gently flexed by hand and by the same person in various directions. Stems were displaced to 30° from the vertical, in different directions, for 5 min a day and for 5 days a week, for 23 weeks. Shoots were then removed by cutting stems at the soil surface and stem length and basal diameter were measured. Whole shoot dry biomass was determined by drying at 65°C for one week.

### Measurement of Root Architecture

An aluminium frame was built to fit over the pots [40] so that the X, Y and Z spatial coordinates of any point in the container could be determined. The substrate was removed progressively with hand tools up to the apex of the root. Spatial coordinates and topology of the origin of all root axes and the end of all root segments, were measured by hand and recorded simultaneously with root diameter in a multitree graph (MTG) format file [41] according to [9]. Root diameter was measured using a pair of vernier callipers. The root segments are defined arbitrarily to take into account branching and changes in root direction or diameter taper.

Roots with a basal diameter less than 0.7 mm were not measured in this way, but the number and mean length of these “additional fine roots” borne by each root segment were noted. The position of nodules was not recorded. The taproot (order 1) is the largest root which continues vertically from the stump or root bole. The whole root dry weight of each seedling was then determined after drying at 65°C for one week.

### Architectural Data Analysis

The characteristics of trees, axes and root segments were computed in the same way as that described in [42], using the AMAPmod software (freely available for Linux and Windows platforms: www.cirad.fr and ftp.cirad.fr/pub/amap/AMAPmod See http://openalea.gforge.inria.fr (Pradal et al. 2008)) [43]. Additional computations, statistics and graphs were produced using the R open statistical package (R Core Team 2012. R: A Language and Environment for Statistical Computing}, R Foundation for Statistical Computing, Vienna, Austria, AMAPmod and R functions for root architecture analysis available from the first authors). The allocation to roots was analysed as a “root partitioning coefficient” (RPC – see list of abbreviations table S1) or root mass fraction. Root length was computed and included the length between the center of the mother segment of the root and daughter root base, i.e. adding the radius of the mother root, thus accounting for primary growth of roots. The mean root diameter (*meandiam_root_*) was computed considering the root as a cylinder:



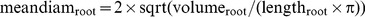
(1)Where volume_root_ is root segment volume and length_root_ is root segment length. The proximal root taper was computed as the % of diameter decrease per cm of root length on the first 3.5 cm root length. Root density was obtained by dividing root dry weight by total root volume. Apical unbranched length is the length between the last branch and the apex of the root.

**Figure 1:**
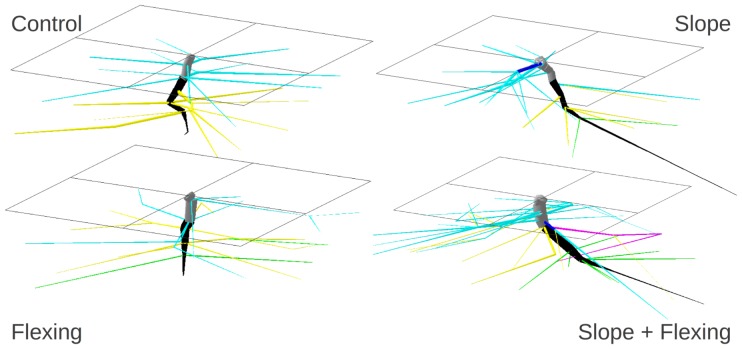
3D reconstruction of one “average” root system per population, chosen from the PCA. (a) control tree n° 24 (b) Slope at 45° tree n° 7 (c) flexed tree n°29 (d) Slope+flexed tree n°19. Segments were coloured as a function of their compartment: grey = (1) stump, black = (2) taproot, dark blue = (3) zone of rapid taper (ZRT), light blue = (4) horizontal shallow roots beyond ZRT, green = (6) deep roots, yellow = (7) intermediate depth roots and magenta = (8) oblique roots. Size is arbitrary but proportional. The black frame is the container wall (0.3×0.3 m width) and the soil surface. North/Upslope is on the left.

Root segments were classified into eight compartments [17], i.e. (1) stump, (2) taproot, (3) zone of rapid taper (ZRT), (4) horizontal shallow roots beyond ZRT, (5) sinkers, (6) deep roots, (7) intermediate depth roots and (8) oblique roots. The initial branching point on the 1^st^ order root was used to classify the lateral roots as a function of their length to the collar. The distance to the soil surface limits between “shallow”, “intermediate depth” and “deep” roots were set to −35 and −70 mm respectively. The limits between horizontal, oblique and vertical roots were 30° and 60° towards the soil surface respectively (figure S1). Root systems had a very low volume of sinkers (0.4%) and oblique roots (0.9%) (Fig. 1), so these compartments were respectively pooled to the taproot and intermediate depth root compartments.

The following adaptations to the computation were made to cope with the small tree size, growth on a slope and the specificities of the measurement method:

1. Spatial analysis was made in the same way for all trees, using X and Y axes parallel to the upper border of the container (i.e. parallel to the soil surface) and a Z axis perpendicular to X and Y and passing through the collar of the tree. The slope containers were all inclined towards the south. Therefore, X is both the north for all trees and the upslope direction (0° azimuth) in tilted pots.
2. Most of the roots were straight, but those which had reached the wall or the bottom of the pot generally followed the wall. To remove this artefact, such roots were virtually extended in the direction the root was growing before it attained the edge, keeping the same segment lengths (see [42]).
3. The angle to the soil surface of the taproot was computed using the line running between the collar and the point where the taproot crosses the “deep root limit” i.e. 70 mm depth [17](figure S1).
4. The mean segment length was large compared to the root system size. Therefore, according to [33], each root segment was divided into 10 mm long virtual segments which were used in the spatial distribution analysis.
5. The stump and taproot of seedlings grown on slopes were 25% more inclined than in the control trees. Therefore, the lower stump limit was computed by using a standard stump length of 45 mm for all trees, instead of a standard stump depth. Because the stump makes no active contribution to tree stability, it was not used in total root volume computations [44]. In the compartment and circular distribution computations, radial distance and azimuth of a segment were not computed relative to the collar position, but relative to the end of the taproot segment which bears the corresponding root arborescence. This “relative radial distance” was used to define the limit between the ZRT and horizontal surface root beyond ZRT, using the 10 mm long virtual segments. The azimuth of axes is computed at the point at 1 cm of the axis base. Stump and taproot are excluded from circular distribution analysis, as they are considered to be the centre of the root system.
6. When specified “additional fine roots” were included in the root length and root number analysis.
7. The root directional deviation (RDD) was computed for each root according to [15] as being the absolute change in root azimuth angle relative to the taproot, between a point at 10 mm from the root origin on the mother root and the root tip. To check for straightness in three dimensions we computed “root winding” which is the length of the root divided by the distance between the root base and tip.
8. The topological index q_b_ was computed according to [10]. q_b_ quantifies the position of an arbitrary binary tree between a wholly dichotomous pattern (q_b_ = 0) and a wholly herringbone pattern (q_b_ = 1). q_b_ is based on the sum of all path lengths from the collar to the exterior links. To check for the circular heterogeneity of branching pattern, we could not use q_b_ because an arborescence can be shared between several sectors. Instead we used simply the mean branching order [45]:
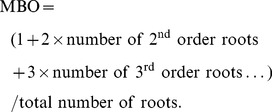
(2)
9. We computed three fractal branching parameters [6], [46]:
  the scaling parameter p_branch_ characterizes the tapering by branching, i.e. it is the cross sectional area (CSA) of the root before the branching point divided by the sum of all root CSA after the branching point. p_branch_ is >1 when tapering occurs.
  the tapering between branching points p_within_,
  the allocation parameter q is the share of the largest root segment after branching in the sum of CSA after the branching point:

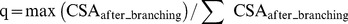
(3)q is close to 1 in a herringbone branching pattern and 0.5 in a dichotomous branching pattern.

The root diameter on the main axis was measured just before the branching point, but not directly after, therefore, if the main root segment after the branch was longer than 2 cm, the CSA of the parent axis 2 cm after the branching point - obtained by linear interpolation - was used in the computations.

### Statistical Analysis

For each of the biomass and architectural parameters, a three way analysis of variance was used to test the Slope and Flexing effects on the Y character; with the Block effect also included (each factor has two levels):
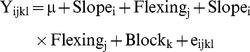
(4)


Y_ijkl_ is the value for the the tree l in level of Slope i, level of Flexing j and Block k. μ is the general mean, e_ijkl_ is the residual error. In the case of non-normal residuals (tested with the Shapiro test), variables were transformed to obtain a normal distribution. The corresponding significance tests of factors were reported in the tables, but the % variation in the value of each combination of treatments with regard to the control was computed from the untransformed variable.

According to [17], to test for circular heterogeneity, three discontinuous slope oriented sectors were defined, upslope (us = 0°±45°), perpendicular to slope (pp) and downslope (ds = 180°±45°). The taproot was discarded for this analysis. To test the “sector” factor (i.e., at least one of the three sectors is different from the others), a simple mixed model with the sector as fixed factor and the tree as random factor was used, separately for each combination of treatment:
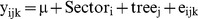
(5)


The significance of differences between sectors was computed using their contrasts. An even distribution results in 25% root volume, root length and number in the upslope and in the downslope sector. The reinforcement in volume of upslope sector is for example expressed as: volumeReinforcementUpslope = (relativeVolumeUpslope – 25%)/25%.

A principal component analysis (PCA) of the 34 trees was made using 17 variables on the centred and scaled values to assess the overall grouping of individuals with regard to these variables. The variables were selected so as to represent the main features of the seedlings.

## Results

### General Tree and Root System Characteristics

**Figure 2:**
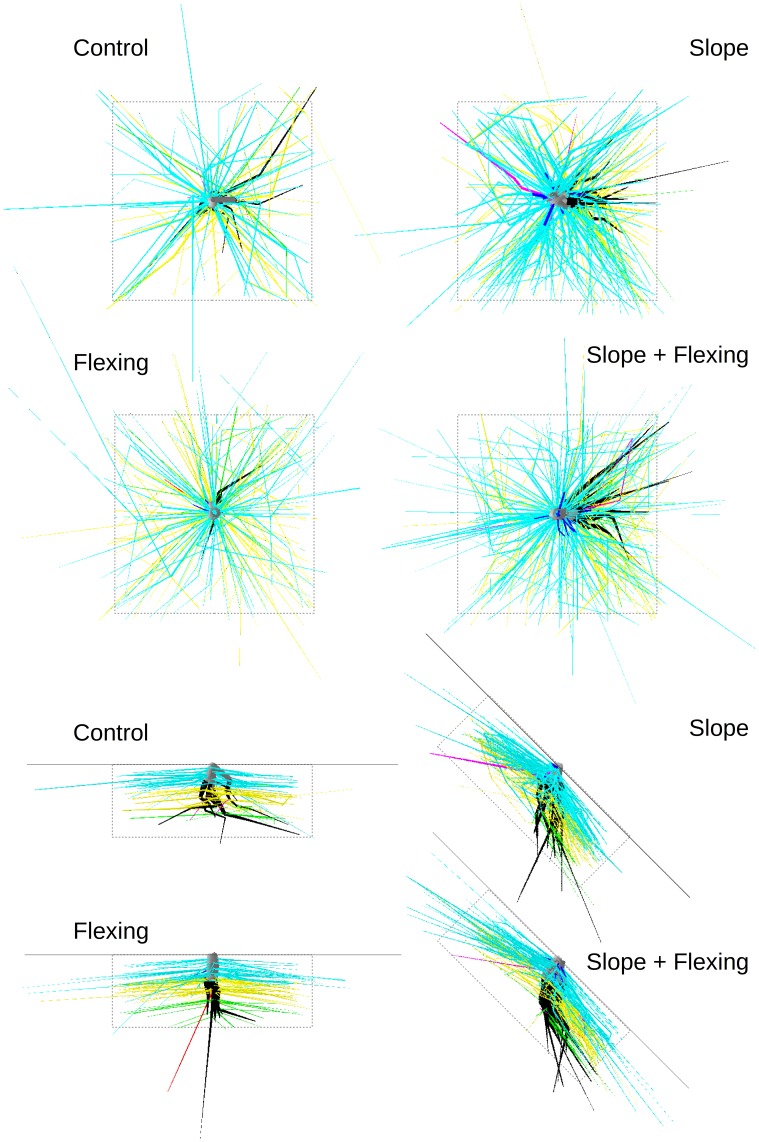
Overplotting of all root systems of each treatment for exploratory data analysis. Colour coding according to Fig. 1. North/Upslope is on the left. Above: top view, perpendicular to the soil surface. Below, side view, perpendicular to the slope. The black line below is the soil surface. The grey dashed frame is the container side and bottom wall (0.3×0.3×0.11 m).

The total seedling biomass was doubled in both the slope (no flexing) and flexed trees when compared to the control (Table 1). Slope+flexed seedlings had almost three times greater biomass than control (47 g vs. 17 g) because of a negative interaction (−34%) between the two factors. Similarly, each treatment resulted in approximately 30% increase in both stem length and collar diameter and a doubling of total root length. Flexing resulted in a 6% increase in stem length/diameter ratio (Fig. 2).

**Table 1:** Shoot and root characteristics of the seedlings.

Variable	Unit	Controlmean(n = 7)	Controlsd	Slopemean(n = 9)	Flexingmean(n = 8)	Slope×Flexingmean(n = 10)	Block	Correlationwith d0
Root+shoot dry weight	g	17.5	4.99	36****	34.3****	46.9		0.96****
Stem length	cm	94.3	8.72	130****	124****	142**		0.94****
Collar diameter	cm	1.01	0.152	1.4****	1.4****	1.7		1****
stem length/collar diam	/	94.4	5.2	93.7	88.7***	83.5		−0.83****
Shoot dry weight	g	14	4.42	29****	25.6****	34.9		0.95****
**Root traits**								
Root dry weight	g	3.49	0.674	7.03****	8.71****	11.9		0.91****
RPC (biomass)	%	20.3	2.5	19.7	25.5****	25.5	**	0.35*
RPC without 1^st^ order root	%	8.27	3.59	9.59	6.67*	6.82		−0.26
Tap root PC	%	2.96	2.38	2.34	5.02****	6.54		0.52***
Stump PC	%	9.12	2.51	7.62*	13.9****	12.1	*	0.23
Max. radial distance	cm	21.8	4.95	25.2	24	27.3		0.28
Max. depth	cm	−10.2	2.01	−13.5	−12.5	−13		−0.24
Overall root length^iafr^	cm	248	119	475**	473**	725		0.69****
1^st^ order root length	cm	16.9	6.81	19.6**	14	22.6		0.43**
SRL 1^st^ order root	cm/cm^3^	4.11	1.5	2.48*	1.66****	1.44		−0.69****
SRL lateral roots^iafr^	cm/cm^3^	88.4	41.3	56.2*	147****	123		0.19
Mean root tip diameter	cm	0.065	0.011	0.07	0.049**	0.056		−0.07
Root density	g/cm^3^	0.528	0.235	0.422**	0.781**	0.564		−0.1

iafr = including additional fine roots.

PC: partitioning coefficient.

RPC: root partitioning coefficient. Unless mentioned, RPC is computed from the volume and not the dry-weight.

SRL: specific root length, i.e. root length/root volume.

Columns 5 to 7: means of each treatment and level of significance of the corresponding factor or interaction: *, <5%; **, <1%; ***, <0.1%; ****, <0.01%.

The root partitioning coefficient (RPC) increased by 25% in flexed plants compared to controls. However, when all 1^st^ order roots (stump+taproot) were excluded from the ratio, flexed plants had a 20% lower RPC. Conversely, if only the taproot was taken into account, RPC was 71% higher in flexed trees compared to controls. If only the stump was taken into account, slope trees had a 17% lower RPC whereas flexed trees possessed a 50% higher RPC (Table 1).

The slope×flexed interaction was never significant in the above mentioned variables, except for those related to the size of the trees. The block effect was never significant in any variable studied, except for RPC, which could not be explained.

### Root Branching and Topology

**Figure 3:**
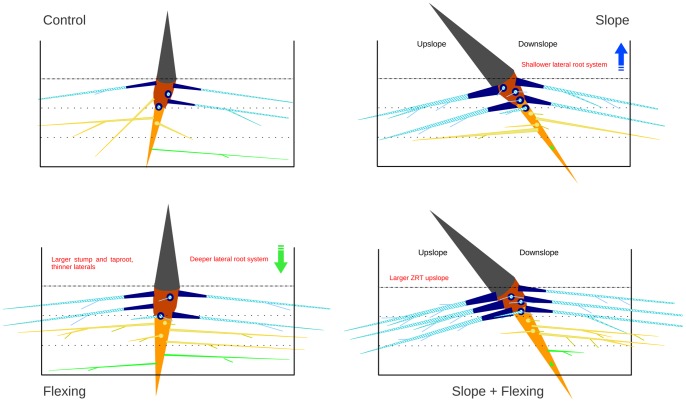
Schematic representation of 26 week old *Robinia pseudoacacia* root systems grown in containers. Colour coding according to Fig. 1. Lateral roots growing perpendicular to the slope direction are shown as dots on the taproot. Root characteristics (e.g. root number, root size) are the average in each group.

**Figure 4:**
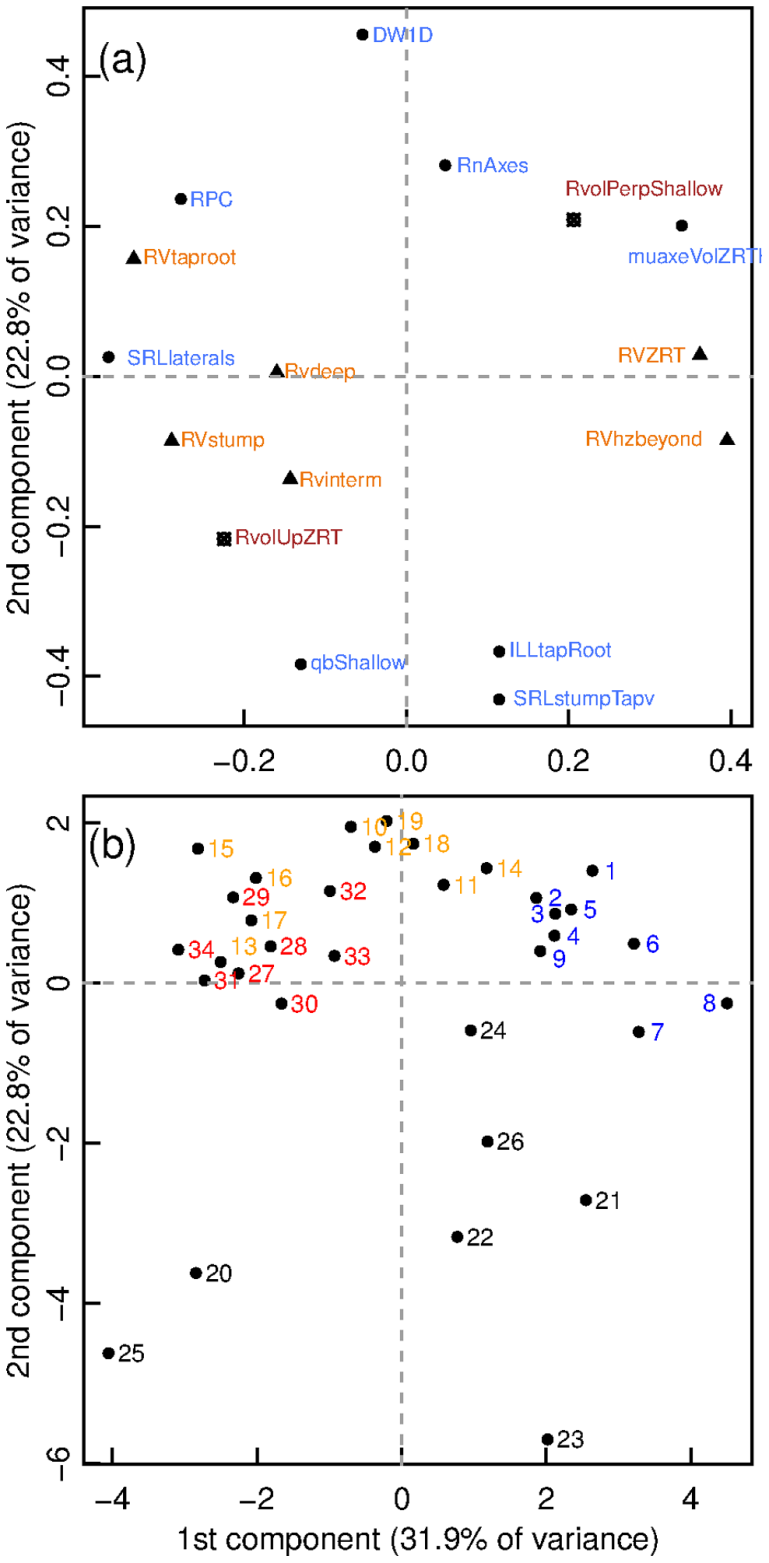
Principal Component Analysis (PCA) of tree characteristics. Scores for PC1 and PC2. (a) Loadings for the 16 original variables. Blue: General tree and root system characteristics and branching variables. RPC = root partitioning coefficient, DW1D = cubic root of seedling dry weight, RnAxes = number of root axes divided by root dry weight, muaxeVolZRT = mean axis volume in the ZRT, SRLaterals = specific root length of laterals, qbShallow = Qb in the shallow root compartment, ILLtapRoot = interlateral length on the taproot, SRLstumpTapv = specific root volume of the first order root. Orange: relative root volume (RV) by compartment; RVhzbeyond is shallow root beyond ZRT, RVtaproot is for the taproot, RVdeep is for deep roots, RVstump is for the stump, RVinterm is for intermediate depth roots and RVZRT is for the ZRT relative root volume. Red: circular distribution of root volume: RvolUpZRT is the volume of root in the ZRT upslope (or north) divided by the whole volume in the ZRT. RvolPerpShallow is the proportion of perpendicular to slope (or east and west) shallow roots out of the total shallow roots. (b) Loadings for the 34 trees of the sample. Black = control; blue = slope; red = flexed; orange = slope+flexing.

The mean total number of roots was significantly greater in both slope and flexed trees compared to control trees, but no differences were found in specific root number (Table 2 and Figs. 1 to 4). Shallow root mean length was 30% greater in flexed trees. Shallow root mean diameter was 37% greater in slope trees and 15% smaller in flexed trees compared to control trees. Shallow root taper was 19% lower in slope trees. This increase resulted in a doubled mean volume in slope trees but a 17% lower mean volume in flexed trees. Intermediate depth roots in flexed trees were 16% thinner resulting in a 40% lower individual volume. Flexed trees also had 20% lower relative volume in 3^rd^ order roots. 1^st^ order roots in the slope trees were 20% less tapered than in control trees. The mean proximal taper of 2^nd^ order roots and deep root individual root dimensions were not significantly different from the control in any treatment.

**Table 2:** Branching characteristics.

Variable		Unit	Controlmean	Controlsd	Slopemean	Flexingmean	Slope×flexingmean	Correlation with d0
Root number	total iafr	n	20.3	8.6	48**	45.1**	67.6	0.64****
SRN (Root number/rootDW)	total iafr	n/g	5.64	1.86	7.25	5.07	5.68	−0.08
Mean axis length	horizontal surface	degree	13.9	4.54	15.9	18*	17	0.46**
Mean axis length	Intermediate depth	degree	14.2	3.95	14.4	15.6	14.4	−0.01
Mean axis length	deep roots	degree	14.9	4.38	14.6	15.1	15.3	0.05
Mean axis diameter	horizontal surface	cm	0.124	0.03	0.17****	0.106****	0.128	0.15
Mean axis diameter	Intermediate depth	cm	0.119	0.03	0.122	0.1*	0.104	−0.07
Mean axis diameter	deep roots	cm	0.102	0.04	0.0932	0.09	0.0914	0.03
Mean axis volume	horizontal surface	cm^3^	0.2	0.13	0.405***	0.167**	0.26	0.3*
Mean axis volume	Intermediate depth	cm^3^	0.205	0.12	0.178	0.127*	0.134	−0.2
Mean axis volume	deep roots	cm^3^	0.137	0.13	0.106	0.0973	0.0931	−0.04
Relative root volume	order 3	%	6.36	4.33	9.53	5.05*	5.86	−0.16
Mean axis taper at 3.5 cm	order 2 on stump	%diam/cm	4.14	0.98	3.68	3.56	3.38	−0.5**
Mean axis taper at 3.5 cm	order 2 below stump	%diam/cm	3.89	1.13	3.97	3.86	3.78	−0.12
Mean axis taper	order 1	%diam/cm	6.16	2.55	5.04***	7.69	4.35	−0.42**
Mean axis taper	order >1	%diam/cm	4.75	0.71	4.99	4.67	5.07	0.12
q_b_	total iafr		0.576	0.16	0.277****	0.382*	0.246	−0.46**
q_b_	shallow roots iafr		0.674	0.25	0.232****	0.339**	0.195**	−0.56***
q_b_	intermediate depth iafr		0.531	0.4	0.766	0.527	0.396	−0.11
q_b_	deep roots iafr		0.613	0.39	1	0.829	0.778	−0.02
Mean p_branch_	stump		1.2	0.2	1.05**	1.23	1.09	−0.2
Mean p_branch_	taproot		1.42	0.31	1.31	1.87**	1.59	0.05
Mean p_branch_	laterals		0.985	0.24	0.981	0.658****	0.739	−0.44**
Mean p_within_	stump	% cm/cm	9.16	13	8.98	7	3.97	−0.19
Mean p_within_	taproot	% cm/cm	24.6	19.4	16.6*	24.9	15.4	−0.38*
Mean p_within_	laterals	% cm/cm	4.96	1.02	6.48	4.23	4.2	−0.28
Mean q	stump		0.781	0.17	0.841	0.926***	0.933	0.6****
Mean q	taproot		0.743	0.08	0.8**	0.791**	0.903	0.57***
Mean q	laterals		0.666	0.09	0.665	0.504****	0.551	−0.34*
Mean inter-lateral length	stump iafr	cm	0.568	0.36	0.364	0.394	0.328	−0.5**
Mean inter-lateral length	taproot iafr	cm	3.93	2.95	1.73	1.1*	1.65*	−0.45**
Mean inter-lateral length	order II iafr	cm	2.87	1.45	1.26	1.11*	1.48**	−0.33*
Mean apical unbranched length		cm	12.7	3.37	13.5	14.5	13.7	0.25
Mean branching angle	order 2 on stump	degree	82.2	8.27	83.2	85.3	82.4	0.14
Mean branching angle	order 2 below stump	degree	87.2	10.7	72.1***	85.7	73.2	−0.47**
Taproot angle toward soil surface	taproot	degree	−71	18	−53.4****	−83.6*	−59.3	0.13
Mean root angletoward soil surface	horizontal order2 on stump	degree	−10.8	10.7	−12	−5.79*	−7.98	0.24
Mean root angletoward soil surface	horizontal order2 below stump	degree	−4.09	3.47	−9.88***	−4.34*	−6*	−0.05
Mean absolute RDD	order 2	degree	11.8	8.71	7.95	5.91	6.3	−0.3*
Mean axis winding	order 2 on stump	degree	105	5.09	104	104	114	0.31*
Mean axis winding	order 2 below stump	degree	105	9.5	102	101	101	−0.29*

iafr = including additional fine roots.

SRN = specific root number.

The block effect was significant only for q_b_ in deep roots (**). Same design as for table 1.

The topological index for the entire root systems indicated that control trees were moderately herringbone (q_b_ was close to 60%) (Table 2). Slope trees were 50% more dichotomous and flexed trees were 35% more dichotomous than control root systems, with additive effects between slope and flexing (Table 2). q_b_ differed only in shallow roots with a 66% increase in flexed and a 50% increase in slope trees, but the factors were only partially additive (Table 2).

The mean distance between laterals along the stump was not significantly influenced by the treatments, but this distance was 60% smaller along the taproot and 2^nd^ order roots in flexed trees but it was not the case in the slope+flexed trees. There was no effect of treatments on the apical unbranched length (Table 2).

### Fractal Branching Parameters

In control seedlings, tapering through branching was average on the stump (p_branch_ = 1.2), high on the taproot (1.43) and negligible on lateral roots. Mean p_branch_ was 13% lower in the stump in slope trees and 30% higher in the taproot but 33% lower in lateral roots of flexed trees (Table 2).

Tapering between branching points was high in the taproot of control trees. p_within_ differed from the control only in the taproots of slope trees (−33%) (Table 2).

In control trees, q averaged 78% in stump, 75% in taproot and 66% in laterals, intermediate between a herringbone and a dichotomous pattern. Mean q was +7% in the taproots of slope trees. In flexed trees, mean q was 19% larger in the stump, 6% greater in the taproot and 25% smaller in laterals roots (Table 2).

### Root Branching Angle and Angle toward the Soil Surface

The mean branching angle of 2^nd^ order roots was smaller beneath the stump in slope trees only (Table 2). The angle towards the soil surface of taproots in control seedlings averaged only 71°, compared to the taproots of flexed plants (84°). Slope seedlings had taproots which possessed a smaller angle towards the soil surface in the slope direction only (slope = −55.0°; slope+flexed = −60.4°, which corresponded to an angle of 80° and 75° toward the horizontal plane, in the upslope direction, respectively - Fig. 3).

However, the mean angle towards the soil surface of 2^nd^ order roots was smaller in shallow roots of flexed (−5.8°) compared to control trees (−10.8°) and greater in roots below the stump in slope trees (−9.9°) and flexed trees (−4.34°) compared to control trees (−4.1°). A low RDD, or root winding, in all treatments indicated very slight root reorientations (Table 2).

### Root Volume, Length and Number in Compartments

In the control trees, the stump represented 45% of the total root volume and all the 1^st^ order roots (i.e. stump+taproot) made up 60% of the total root volume (Table 3). When the stump was excluded, horizontal shallow roots comprised 45% of the total root volume, with the remaining volume allocated to the taproot (25%) and the horizontal intermediate depth roots (25%). Deep root volume was only 2.5% (Table 3).

**Table 3:** Root volume, length and number proportion in the six compartments.

Variable	unit	Controlmean	Control sd	Slope mean	Flexing mean	Slope×flexing mean
	Relative volume					
(1) Stump	%	45	12.9	38.9*	54.3**	47.4
	Relative volume, stump excluded					
(2 & 5) Taproot & Sinkers	%	26.7	21.7	20.2	44****	49.8
(3) ZRT	%	11.6	9.04	20.2**	9.18*	12.6
(4) Horizontal shallow beyond ZRT	%	32.7	24.4	41.7	20.4**	23.6
(8 & 9) Intermediate & Oblique	%	26.5	25.1	15.9*	21.7	12.4
(7) Deep roots	%	2.54	3.55	1.31*	4.68	1.5
	Relative length^iafr^					
(3 & 4) Horizontal shallow	%	26.7	21.7	20.2	44****	49.8
(8 & 9) Intermediate & Oblique	%	11.6	9.04	20.2**	9.18*	12.6
(7) Deep roots	%	32.7	24.4	41.7	20.4**	23.6
	Relative number^iafr^					
(3 & 4) Horizontal shallow	%	47.9	27.5	63.9*	44.2	58.4
(8 & 9) Intermediate & Oblique	%	31.4	24.1	24.7	37.6	27.6
(7) Deep roots	%	9.8	15.8	2.81	8.6	4.4

iafr = including additional fine roots.

The stump is only included in the first line. The ZRT and horizontal shallow beyond ZRT compartments are pooled together in a “horizontal shallow” root compartment for the root length and number proportions. Additional fine roots are included in the length and number proportions. The block effect was never significant. Same design as for table 1.

Mean stump volume was 14% smaller in slope trees and mean volume of intermediate depth roots and deep roots was 40% lower whereas the mean volume of the ZRT was 75% higher (Table 3). Conversely, flexed trees had a greater proportion of volume allocated to the stump (+20%) and taproot (+67%) and a larger horizontal shallow roots relative length (+65%), but 22% less volume in the ZRT and 38% less in the horizontal shallow roots beyond the ZRT. Root length (+23%) and number (+33%, including additional fine roots) differed from the control only in horizontal shallow roots of slope trees (Table 3).

### Circular Distribution of Roots

No differences in the circular distribution of root volume, number or length were found in seedlings on 0° slopes, regardless of flexing treatment (data no shown). In whole root system and in the ZRT, shallow roots beyond ZRT, intermediate depth roots and deep roots, slope seedlings had 2/3 or more of their root volume, number and length situated in the sector perpendicular to the slope direction (Table 4). Differences between upslope and downslope sectors were significant only with regard to intermediate (8% upslope) and deep roots (there were no deep roots upslope - Table 4). The number of 2^nd^ order roots branching from the stump showed the same circular pattern as that of shallow roots. Downslope shallow roots were more herringbone (+30% for MBO) because only two seedlings developed 3^rd^ order roots in this sector (Table 4). No significant differences between treatments and controls were found with regard to either mean individual root dimensions or mean angle towards the soil surface. Mean branching angle of laterals on the stump was 100° upslope, 80° perpendicular to slope and 67° downslope, as the taproot was close to the vertical whereas the laterals were parallel to the soil surface (Table 4).

**Table 4:** Circular distribution of various root system characteristics in the three discontinuous slope oriented sectors.

		Slope			Slope+flexed			
	*P*	us	pp/2	ds	*P*	us	pp/2	ds
	***%***				***%***			
	**Root volume (% by sector)**							
Total	**0.092**	19.3b	33.2a	14.3b	**11**	29.4	23.7	23.2
ZRT	**0.29**	19.8b	33.5a	13.1b	**0.11**	36.6a	24b	15.4b
Horizontal beyond ZRT	0.74	19.1b	34.2a	12.5b	2.4	32.3a	24.4ab	19b
Intermediate depth	**2.3**	8.76b	32.1a	27.1a	**12**	16	25	34
Deep roots	**0.0012**	0c	43.1a	13.8b	**5.5**	4.55b	29.3ab	37a
	**Root length (% by sector iafr)**							
Total	**0.014**	17.3b	33.5a	15.7b	**6.3**	28.9	22.8	25.5
Shallow	**0.17**	20.4b	33.7a	12.2b	**0.58**	34.1a	23.2b	19.5b
Intermediate depth	**0.85**	8.36b	32a	27.6a	**12**	17.4	25.1	32.4
Deep	**0.068**	0c	41.7a	16.6b	**13**	8.56	26.9	37.7
	**Root number (%n by sector)**							
Total^iafr^	**0.058**	16.7b	34.1a	15b	**0.16**	32.9a	22b	23.1b
Shallow^iafr^	**0.57**	20.3b	34.2a	11.3b	**0.058**	38.3a	22.1b	17.5b
Intermediate depth^iafr^	**0.31**	6.74b	32.5a	28.2a	**51**	20.7	24.8	29.6
Deep^iafr^	**0.49**	0b	41.7a	16.7b	**20**	10.9	25.9	37.2
Order II on stump (1)	**2.4**	18.9b	32.6a	15.8b	**48**	24.9	26.5	22.1
Order II below Stump (1)	**1.8e**−**09**	0.855c	34.9a	29.4b	**0.25**	14.2b	26.9a	32a
	**MBO (iafr)**							
Total	**0.32**	1.43a	1.3a	1.14b	**15**	1.39	1.3	1.3
Shallow	**0.45**	1.43a	1.35a	1.11b	**44**	1.41	1.34	1.36
Intermediate depth	**NA**	NA	NA	NA	**91**	1.24	1.22	1.21
	**SRL (cm/cm^3^)**							
Shallow	**22**	40	36.8	50.1	**1.3**	53.2b	79.3ab	103a
Intermediate depth	**33**	82.8	89	122	**57**	106	119	132
	**Mean axis diameter (cm)**							
Shallow	**61**	0.17	0.173	0.162	**0.32**	0.157a	0.121b	0.122b
Intermediate depth	**70**	0.116	0.125	0.119	**50**	0.109	0.103	0.1
	**Mean axis length (cm)**							
Shallow	**55**	15.9	15.3	16.6	**41**	19	16.5	18.8
Intermediate depth	**20**	13	14.8	12.7	**14**	12.9	15	14.5
	**Mean axis volume (cm^3^)**							
Shallow	**81**	0.385	0.406	0.351	**14**	0.411	0.214	0.328
Intermediate depth	**62**	0.169	0.19	0.149	**54**	0.118	0.147	0.123
	**Mean axis taper at base (%diam/cm)**							
Order II on stump	**88**	4.1	3.71	4.05	**77**	3.39	3.36	3.65
	**Mean branching angle (degrees)**							
Order II on stump	**1.9**	99.8a	79.7b	66.9b	**0.063**	90.4a	85.8b	60.7c
Order II below Stump	**NA**	NA	NA	NA	**0.00021**	114a	75b	51c
	**Mean angle toward soil surface (degrees)**							
Shallow	**89**	−11.6	−11.5	−11.6	**0.0034**	−12.9a	−6.21b	−5.49b
Intermediate depth	**17**	−6.28	−9.43	−10.3	**84**	−6.83	−6.12	−6.79

(1) For the 2^nd^ order additional fine roots, the sector to which they belonged, with regard to slope orientation, was not recorded. Additional fine root number on the stump averaged 4.4 (80% of the number of larger roots) in the slope treatment and 3.9 (45% of the number of larger roots) in the slope+flexing treatment. These values reached 3, 36%, 4.8 and 67% for the number of 2^nd^ order additional fine roots branching below the stump respectively.

“us” = upslope, “ds” = downslope, “pp” = discontinuous sector perpendicular to slope. For the “pp” sector, when the variable is a root volume, length or number, the value in the table and for the statistical analysis was divided by 2. P values (%) are from the mixed model test of fixed factor “sector;” “tree” is the random factor. Data not shown for control seedlings, or seedlings grown with flexing on a 0° slope, as no significant differences were found, except for root length in the shallow root compartment (P = 2.9%) in control trees. When the sector effect is significant, sectors with the same letter in superscript are not significantly different. Data for the deep root compartment were often not available (NA) because few trees had roots in this compartment.

In slope+flexed seedlings no significant differences were found with regard to the all roots distribution, except for root number which had a 32% increase in the upslope sector (Table 4). The ZRT and horizontal beyond ZRT compartments possessed a 46% and 29% reinforcement of root volume in the upslope quadrant respectively, at the expense of the downslope quadrant. Shallow root length and number also augmented in the upslope quarter by 36% and 53%, respectively (Table 4). Conversely, root volume and root number were lower in upslope deep laterals and in the upslope 2^nd^ order root branching below stump, compared to those downslope (Table 4). The mean root diameter of shallow roots was 30% larger and SRL 33% smaller upslope, compared to all other sectors. The other root dimension and topology variables were uniformly distributed. The mean root angle towards the soil surface in perpendicular and downslope roots was significantly smaller (−6°) compared to both the upslope and shallow roots of the slope trees (approximately −12°) (Table 4). Therefore, along with differences in taproot orientation, this result explains why 2^nd^ order root branching angles in slope+flexed seedlings were smaller than in slope trees.

### Multivariate Analysis

The PCA highlighted the intensity in grouping of the four tree types (Fig. 4). Data from control seedlings scattered much more than from the other seedlings, and were distinctly grouped towards high negative loadings for the second component (PC2) associated with poor development (low biomass “DW1D”, low root number “RnAxes”, greater shallow roots topological index “qbShallow”, higher taproot SRL “SRLstumpTapv” and larger inter-lateral root length on taproot “ILLtapRoot”. Slope seedlings were strongly grouped toward large positive loadings for the first component (PC1), corresponding to a large relative volume in shallow roots (“RVZRT and “RVhzbeyond”) and a large mean axis volume in the ZRT (“muaxeVolZRT”). Flexed trees and two control trees had large negative loadings for PC1, associated with a high SRL without taproot (“SRLlaterals”), high stump and taproot relative volume (“RVstump” and “RVtaproot”) and high RPC,. The largest slope+flexed trees were localised in between the flexed and the slope trees. The remaining 40% of smaller trees were grouped with the flexed trees indicating a large influence of flexing on a number of traits. Both intermediate depth and deep root relative root volumes (“Rvinterm” and Rvdeep”) contributed little to the two first axes of the PCA, as well as circular distribution variables (“RvolUpZRT” and “RvolPerpShallow”).

## Discussion

### All the Control Trees were Small

Both the slope and flexing treatments increased plant size significantly, augmenting also variables such as number of roots. The poor development of control trees was associated with a higher variability of size and root architecture parameters. These results were not due to differences in soil moisture content. No run-off was observed during watering because the substrate was highly absorbent. At a small scale, a higher water accumulation may have occurred at the deepest point in the rotated containers, downslope, which could partly explain the increased length of taproot in the slope treatment compared to the trees grown on flat ground. Nevertheless, some soil compaction downslope of inclined plants could be seen, which may be due to the consolidating effects of water infiltration downslope. In a similar experiment, when containers were inclined 22° and 45°, total dry biomass of inclined plants increased by 100%, compared to controls [47]. It was also found [36] that biomass was increased in roots and shoots of *Spartium junceum* L. tilted at 45°. It is often held that dynamic mechanical loading negatively influences plant size [24], although [48] reported that wind loaded *Helianthus annuus* L. plants were taller with a higher stem hydraulic conductivity whilst flexed plants were shorter with a lower stem hydraulic conductivity, compared to controls. In a novel experiment, [49] found that seedlings of *Pinus pinaster* Ait. subjected to artificial and repetitive wind loading in the field also had significantly more biomass compared to controls. The mechanism by which mechanical loading increases plant biomass, and presumably vigour, is not known. However, a variety of physiological responses occur within a plant as it moves [21], with as yet not fully understood implications for metabolic pathways and hence growth.

The control trees were smaller and possessed only a thick taproot and only a few branches whereas larger trees from other treatments also possessed 3^rd^ order roots, especially near the soil surface. Hence, root systems from the slope and flexed treatments were more dichotomous, as were also compartments or circular sectors with a small number of roots. Similarly, because of their small size, control trees had a smaller q in the stump and taproot.

Tapering by branching or between branches took place mainly on the only vertical root, i.e. the taproot. Such a rapid decrease of root biomass as a function of soil depth is typical in most plants [50].

### Root Systems Growing on a Slope were Shallow and Asymmetric

Pot and soil geometry may explain several slope effects: taproots grew vertically downwards and each pot was inclined, thus 40% more soil volume was available in the vertical direction beneath the centre of each tree. Additionally the bottom of the container was inclined. Therefore, slope trees had a larger maximal rooting depth and relative stump volume and a smaller taproot SRL, taper and q. Lateral roots remained closely parallel to the soil surface even at 10 cm depth, as also found by [35] who hypothesized that superficial lateral roots responded to a signal e.g. oxygen, light or temperature, which enabled them to grow parallel to the soil surface, regardless of slope angle, but also to deflect if the root came into direct contact with the surface. The root system of slope trees was shallow, with well developed shallow roots at the expense of intermediate depth and deep roots. This result may be explained by the inherent root architectural scheme of these plants e.g. a 4 cm long stump in slope trees bore the same mean number (5.5) of 2^nd^ order axes as a control tree in a 2.8 cm thick soil layer instead of 4 cm. Similarly, deep lateral roots could not develop upslope because as they emerged from the taproot, their growth would be impeded by the base of the pot (Fig. 2).

The predominant overall development of lateral roots in the sectors perpendicular to the slope direction cannot be explained by geometric differences: in trees on slopes, the distance between the stump and pot wall was approximately the same in the four main directions, although the circular distribution of volume and length and number of shallow roots was highly heterogeneous. Stem and crown weight plays an important role in determining the distribution of internal stresses within a mature tree [51]. However, in our study on seedlings, the stems were almost straight, and only a few short branches appeared, therefore, static stem loading would be low compared to the anchorage capacity of the root system. We suggest therefore that the lateral shallow roots are primarily shallow and secondarily plagiogravitropic. Upslope and downslope roots must grow obliquely upward or downward to stay shallow, which likely reduces growth rate. There were significantly more 2^nd^ order roots in the perpendicular to slope sector in slope trees but a uniform circular distribution of 2^nd^ order roots on the stump in slope+flexed trees. This disparity may result from heterogeneous root mortality or late root emergence but was probably not from heterogeneous initial initiation of roots, as flexing took place 3 weeks after germination. Additionally, the circular sector of additional fine roots was not determined.

### In Response to Flexing, Root Architecture was Modified

Although flexing intensity was low, the resulting changes in seedling structure were major. Stem base diameter and taper increased, as has often been reported for many species (see e.g. [24], [52]). An increase in root wood density increased the mechanical resistance of roots, which could also have been achieved with the same construction costs through thickening of roots [53]. An increase in RPC also improved anchorage [17]. Flexed seedlings also had thicker stumps and taproots, at the expense of ZRT, shallow lateral roots and taproot length. Taproots were relatively short because of the small available soil depth. Their larger tapering through branching compared to the control is due to the smaller diameter of lateral roots. The mechanical behaviour of a taproot has been likened to a stake in the ground, held in place by more flexible lateral roots [54]. Therefore, an increase in stem basal diameter as well as thickening of the stump and taproot will augment rigidity in those areas subjected to the greatest mechanical stresses during flexing, and thus will improve anchorage. In a similar experiment, but with *Quercus robur* L. seedlings subjected to directional wind loading in the field, where soil depth was not limiting, [26] showed that lateral root number and length increased, but at the expense of taproot length. However, in trees subjected to e.g. vertical uprooting through grazing, a more efficient design to prevent uprooting would be a longer taproot with little taper and guyed by deep roots, as in *Q. robur* in [31].

Flexed plants possessed more numerous, thinner, longer and straighter lateral roots which had a more homogeneous vertical distribution along the taproot than in control plants. As these roots hold the taproot in position all along its length, deeper lateral roots will move the centre of rotation of the taproot further to the bottom, constraining overall rotation [54]. Lateral roots are held in tension and for the same amount of biomass, thinner roots are more mechanically resistant, because they possess more cellulose, itself being highly resistant to failure in tension [55]. Therefore, flexed plants will be better anchored in the soil. Because laterals were thin, they demonstrated a low tapering by branching and a small q, and q was smaller on the stump and taproot. Flexing did not reinforce ZRT because thinner roots are less resistant in bending.

Changes in the inclination and branching angles in 1^st^ order roots and lateral roots also improved plant anchorage because a vertical taproot with horizontal laterals provide the best mechanical design in the type of soil used [56]. In addition, [31] observed that unidirectional flexing of the stem in sandy loam resulted in more fine roots with branching angles close to 90° with regard to the vertical in *R. pseudoacacia*, and an overall reinforcement of taproot and sinkers in *Q. robur* seedlings.

### Tree Mechanical Design is Modified, Taking into Account Soil Geometry

Slope and flexing factors were additive for most of the root characteristics. A large part of changes due to the factor slope originated from geometrical constraints, and were also found in slope+flexed seedlings. Elsewhere, as flexing had larger effects on root architecture than slope, slope+flexed trees were similar to the flexed only trees, as seen in the PCA, meaning that plants responded to flexing more than they did to slope treatment.

**Figure 5:**
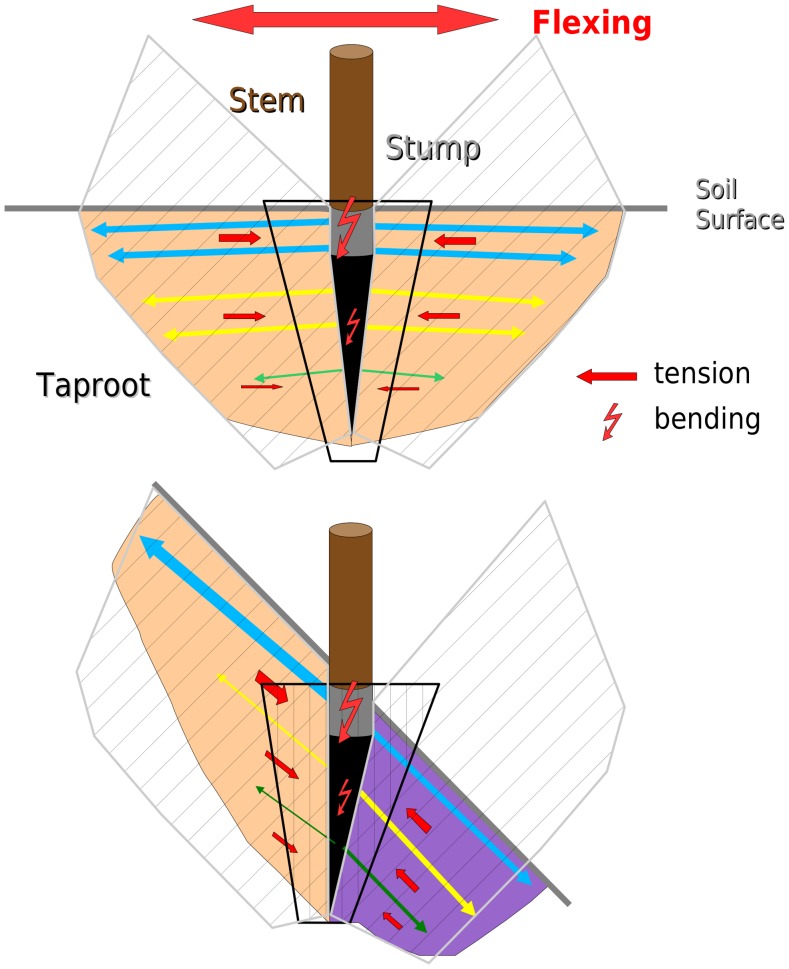
Schematic representation of hypotheses concerning the modifications in response to flexing of seedlings on 0° or 45° slope. All flexed seedlings have a large rigid vertical stump (grey) and taproot (black) and finer lateral roots parallel to the soil surface (blue arrows), analogous to guy ropes around a vertical stake. The 1^st^ order root tapers and lateral roots (yellow and green arrows) become shorter and thinner with depth. The 1^st^ order root undergoes bending, whereas fine roots act in tension. The volume of soil in which the 1^st^ order roots can be potentially embedded is a truncated cone (black vertical hatched zone). The potential volume of soil which can be explored by the guying lateral roots is shown by the grey oblique hatched zones. Above: On 0° slope, most of the hatched zones are filled with soil (orange shading). Lateral roots are both horizontal and perpendicular to the 1^st^ order root. A design with fine and evenly distributed lateral roots is efficient for keeping the stem vertical when lateral forces are dominant. Below: On 45° slope: the above mentioned design is no longer efficient, the resulting root system pattern does not allow for the stump to be held in place, nor for lateral roots to firmly anchor the stump and taproot. As the 1^st^ order root is no longer perpendicular to the soil surface, there is no soil in a large part of the hatched zones downslope and lateral roots are oblique and not perpendicular to the stump and taproot. However, the hatched zone upslope is completely filled with soil (orange shading). Therefore, rotation of the stump is prevented by the thicker upslope shallow laterals which literally hold the stump in place. There is also a progressive shift in the circular distribution of deep laterals downslope as a function of soil depth (violet shading). These deeper roots will help the taproot to be strongly anchored in the soil.

The largest interaction between the two factors could be seen with regard to the circular distribution. As a response to flexing, 2^nd^ order roots were distributed all around the stump to ‘pin’ it in place, but upslope shallow roots were more developed, particularly in the ZRT, likely at the expense of downslope shallow roots. Roots in the upslope sector will provide better anchorage for the stump because (Fig. 5): (1) they will embrace a larger volume of soil between the vertical taproot and the lateral roots parallel to the soil surface. This increase in soil volume will result in a heavier root-soil ball, which will help the plant resist uprooting [57]. (2) Upslope roots also secure the tree stump which would otherwise be more easily rotated downslope, as there is less soil downslope to hold it in place. This geometrical effect thus explains why in winching tests of mature *P. sitchensis*, [58] found that trees were significantly less resistant when winched downslope compared to upslope. In our study, in order to counteract this rotation of the taproot during overturning, deep roots were also well developed downslope, in a soil volume allowing room for growth and thus better anchorage.

In flexed×slope plants, taproots were oriented slightly more upslope and shallow lateral roots growing perpendicular to the slope and downslope had a smaller angle to the soil surface. Therefore, a larger soil volume will be encased by the downslope and perpendicular roots, resulting in a larger root-soil plate downslope, hence increasing root system anchorage. A negative interaction between slope and flexing for q_b_ of shallow roots and for taproot and 2^nd^ order roots inter-lateral length was obtained. It is possible that these interactions between flexing and slope angle were not greater, because certain root traits were confined within given plastic limits. Thus, the interaction of individual root trait reponses to several environmental processes does not equal the sum of trait responses.

Our results are comparable to those found by [37], who found that trees growing in steep elfin forest in Ecuador had a preferential upslope development of roots.

During a winching test or a natural windstorm, the root-soil plate volume is largely composed of the side of the root system held in tension. Thus our results explain why, in winching tests performed up- down- and cross-slope on mature *P. sitchensis* growing on a 30° slope with cross-slope dominant winds, the estimated root-soil plate volume was greatest in trees pulled downslope, compared to those of trees pulled up or cross-slope [58]. However, in the same study, the largest coarse root volume was found on the windward side of the tree, which was perpendicular to the slope direction. But in control trees growing on flat ground, there was more root volume both on the leeward and windward sides [15]. The *P. sitchensis* trees had no taproot which may result in a different mechanical design than in taprooted trees. No architectural analysis was made in this study, therefore, it is not known if a disparity in allocation to different root types occurred.

### Deep Phenotyping Yielded Clear-cut Results

We yielded clear-cut results, largely because in our experiment, all factors were simplified, i.e., we used a homogeneous substrate with direct seeding on a steep slope, as well as simple non directional flexing without airflow. We studied a pioneer species which is likely to possess highly plastic traits. All changes in root architecture could be tracked due to a complete 3D measurement and an in depth analysis. This paper therefore presents a framework for the study of 3D coarse root architecture in all its aspects, i.e., a “deep phenotyping” [13]. Such an analysis would be particularly useful for analysing data from computed tomography (CT) images [13], provided that topology can be extracted from the data. As in [17], the circular analysis of root distribution was adapted to directly reveal changes due to a directional environmental condition.

### Acclimation Takes Place within the Boundaries of Substrate Geometry and the Architectural Model

It has previously been stressed that the architectural model determine the characteristic architecture of the root system in a given plant species and define the limits for plasticity of that species [59]. Our experiment highlights the variability in plasticity of various root architectural traits in response to two environmental factors. The overall root architecture, i.e. a thick, straight, gravitropic taproot with lateral roots parallel to the soil surface had negligible plasticity, therefore, it corresponds to the architectural model [60] for *R. pseudoacacia*.

Differences observed with regard to the inter-lateral length on the stump were related only to tree size (ρ = 0.5). Nevertheless, major plasticity was observed as plants allocated differently resources to various parts of the root system, in particular the diameter and length of laterals versus the taproot, and the circular distribution of overall lateral root development. Even though shallower roots have a significantly higher potential to contribute to plant productivity than deep roots [61], the relative root volume and length of shallow roots varied largely, **e.g.** it was two times lower in the flexed trees compared to the slope only trees. As already shown in mature trees [17], the root architecture of these young trees was modified as an acclimation to MP, but this reaction also took place within the boundaries of substrate geometry and the architectural model of the species.

### Practical Applications

As for trees on flat ground [17], we showed that on steep slopes, trees undergo major acclimation to mechanical perturbation through selective reinforcement of root architecture. It should now be verified with further measurements of root architecture, lateral winching tests and mechanical modelling, if similar results occur in other species of different sizes and in various soil conditions. It is necessary to verify if the observed acclimation and mechanical design is specific to *R. pseudoacacia* seedlings, or if it is a broader phenomenon for trees of diverse root system architectures when grown on steep slopes.

When planting trees on steep slopes in a windy climate, nursery and planting practices should allow for a symmetrical development of shallow roots, so that the optimal development of roots in the direction allowing the best mechanical support can occur.

## Conclusions

In natural environments, many factors such as soil type and heterogeneity, slope angle, water supply, competition and complex wind patterns interact at various levels, therefore it is difficult to characterize the effect of each factor. As a tree grows, it will constantly perform trade-offs to improve performance with regard to e.g. light capture, resource acquisition and mechanical stability within the limits of its architectural model. Tree root systems on steep hillslopes will be highly variable and many types of architecture have been described in the literature, with no one conclusive study of slope effects on root system architecture. Even if it has not been described previously, in any case, woody plants on a steep slope likely have to avoid a downslope displacement of the stump. Therefore, development of larger shallow roots upslope, regardless of whole root system architecture would increase anchorage. An alternative would be the development of a well anchored thick shallow root downhill, acting like a chuck, like in [17] on flat ground.

However, our study goes a long way in quantifying in one typical case, the previously unknown effects of substrate geometry on root architecture, and to differentiate the architectural consequences of inherent root architectural model and root trait plasticity.

## Supporting Information

Figure S1 **Description of angles used in the geometrical analysis of root systems.** Branching angle is the angle between the first segment of the branch and the root segment bearing that branch.
(TIFF)

Table S1 L**ist of abbreviations.**
(DOC)
