# Supplementary figures and images for: Deep Phenotyping of Coarse Root Architecture in R. pseudoacacia Reveals That Tree Root System Plasticity Is Confined within Its Architectural Model
Source: PLoS One. 2013 Dec 27;8(12):e83548. doi: 10.1371/journal.pone.0083548 (PMC3873950; doi:10.1371/journal.pone.0083548)

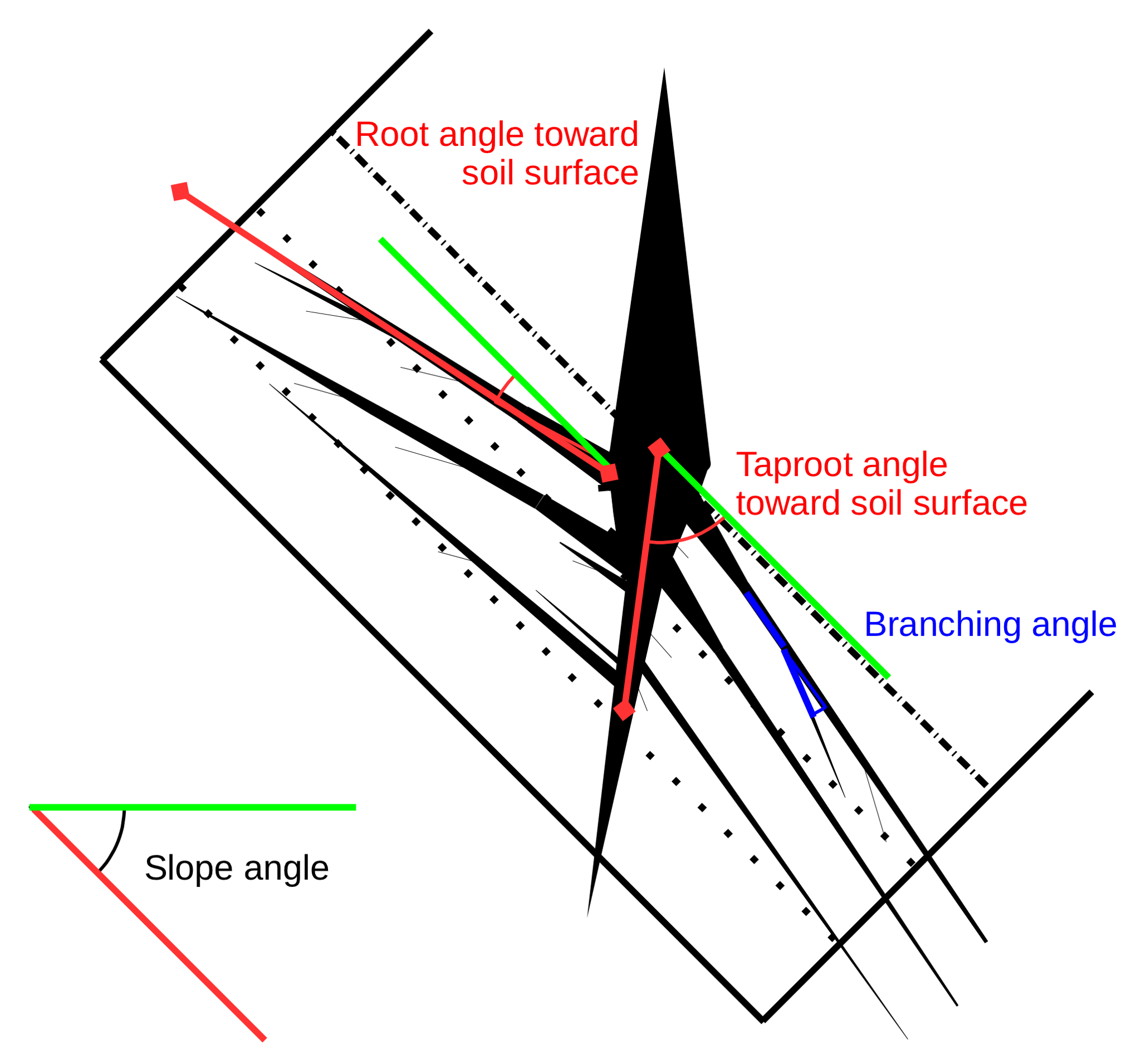

Supplement: Figure S1 — Description of angles used in the geometrical analysis of root systems. Branching angle is the angle between the first segment of the branch and the root segment bearing that branch. (TIFF) [file pone.0083548.s001.tiff]
