# Supplementary material for: Deep Phenotyping of Coarse Root Architecture in R. pseudoacacia Reveals That Tree Root System Plasticity Is Confined within Its Architectural Model
Source: PLoS One. 2013 Dec 27;8(12):e83548. doi: 10.1371/journal.pone.0083548 (PMC3873950; doi:10.1371/journal.pone.0083548)
Supplement: Table S1 — List of abbreviations. (DOC) [file pone.0083548.s002.doc]

table S1 : table of abbreviations

| Abbreviation | Meaning | Reference |
| --- | --- | --- |
| CSA | “Cross sectional area” of root | [57] |
| MBO | “Mean branching order” | [45] |
| PCA | “Principal components analysis” |  |
| q_b_ | Topological index - (wholly herringbone pattern: q_b_ = 1) | [10] |
| p_branch_ | Tapering by branching | [46] |
| p_within_ | Tapering between branching points | [46] |
| q | Allocation parameter | [6] |
| RDD | “Root directional deviation” (azimuth) | [15] |
| RPC | “Root partitioning coefficient” or root volume fraction | [62] |
| SRL | “Specific root length” (root length/root volume) |  |
| ZRT | “Zone of rapid taper” in shallow roots | [63] |
